# Down-regulation of phosphoglucomutase 3 mediates sulforaphane-induced cell death in LNCaP prostate cancer cells

**DOI:** 10.1186/1477-5956-8-67

**Published:** 2010-12-16

**Authors:** Chan-Hee Lee, Soo-Jin Jeong, Sun-Mi Yun, Ji-Hyun Kim, Hyo-Jung Lee, Kwang Seok Ahn, Suk-Hyun Won, Hyun Seok Kim, Hyo-Jeong Lee, Kyoo-Seok Ahn, Shudong Zhu, Chang-Yan Chen, Sung-Hoon Kim

**Affiliations:** 1 College of Oriental Medicine, Kyung Hee University, Seoul 130-701, Republic of Korea; 2 Yonsei University School of Medicine, Seoul 120-752, South Korea; 3 Beth Israel Deaconess Medical Center, Harvard Medical School, Boston, MA 02215, USA

## Abstract

**Background:**

Sulforaphane (SFN) is an isothiocyanate found in cruciferous vegetables that exerts anti-oxidant, anti-inflammatory, anti-cancer and radio-sensitizing activities. Nonetheless, the mechanism responsible for SFN-induced cell death is not fully understood. In the present study, anti-cancer mechanism of SFN was elucidated in LNCaP prostate cancer cells.

**Results:**

SFN exerted cytotoxicity and increased TUNEL positive cells in a concentration-dependent manner in LNCaP cells. Proteomics study revealed that levels of nine proteins including tubulin β-2, phosphoglucomutase-3 (PGM3), melanoma-derived leucine zipper containing extra-nuclear factor, activin A type I receptor precursor, smoothelin-A, KIA0073, hypothetical protein LOC57691 and two unnamed proteins were changed over 8 folds in SFN treated LNCaP cells compared to untreated control. We have further confirmed that SFN reduced PGM3 expression with western blotting and showed that PGM3 siRNA enhanced cytotoxicity demonstrated by cell morphology and TUNEL assays in LNCaP cells.

**Conclusion:**

Taken together, these findings suggest that PGM3 plays a role in mediating SFN-induced cell death in LNCaP cells, and is a potential molecular therapeutic target for prostate cancer.

## Background

Cell death is defined as an irreversible loss of plasma membrane integrity. Historically, three types of cell death have been distinguished in mammalian cells by morphological criteria, namely apoptosis, autophagy and necrosis [1]. Apoptosis represents a major regulatory mechanism that eliminates abundant and unwanted cells during embryonic development, growth, differentiation and normal cell turnover [2,3]. Recently, targeting apoptosis is thought to be a potential therapeutic approach for cancer treatment.

Prostate cancer develops in nearly 30% of all men above the age of 50 years [4] and may metastasize to other parts of the human body, especially bones and lymph nodes. Many chemotherapeutic agents such as Eulexin, Flutamide and Nilandron have been developed for the treatment of prostate cancer. However, undesirable side effects such as urinary incontinence and erectile dysfunction can reduce the therapeutic efficacy of prostate cancer. In this regard, recent reports have reported the possibility to use natural compounds as chemopreventive candidates by inducing apoptotic cell death in prostate cancer cells [5-9].

Sulforaphane (SFN) (Additional file 1: figure S1) is a breakdown product of Glucoraphanin which is the compound present in cruciferous vegetables [10]. Many groups reported that SFN induced apoptosis though activation of caspase-3 and cell cycle arrest in prostate cancer cells [11,12]. However, its exact molecular mechanisms whereby SFN mediates apoptosis are not fully understood. Thus, in the present study, anti-cancer mechanisms of SFN to induce apoptosis were investigated by a comparative proteomic analysis. We here demonstrate that phosphoglucomutase-3 (PGM3) plays a role in the regulation of SFN-induced apoptosis in LNCaP prostate cancer cells.

## Results

### Sulforaphane exerts cytotoxicity and induces apoptosis in LNCaP cells

**Figure 1:**
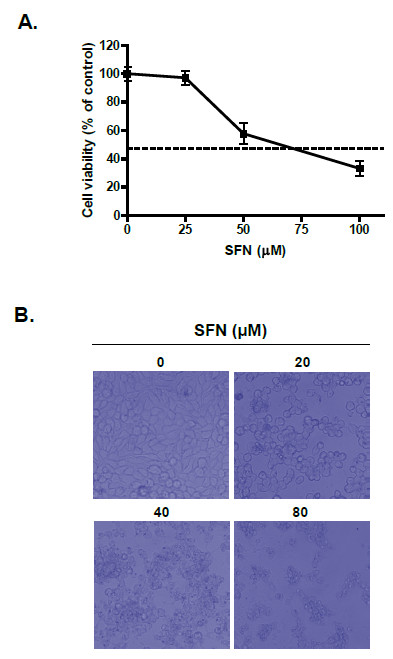
**Effect of sulforaphane on the cell viability in LNCaP cells**. (A) Cells were treated with 0, 25, 50 or 100 μM SFN for 24 h. Cell viability was determined by the XTT assay. Data represent means ± SD. (B) Morphological changes of SFN-treated LNCaP cells by inverted microscopy.

XTT assay was performed to investigate cytotoxic effect of SFN in LNCaP cells treated with various concentrations (0, 25, 50 or 100 μM) of SFN for 24 h. As shown in Figure 1A, SFN exerted cytotoxicity against LNCaP cells in a concentration-dependent manner. To confirm the cytotoxicity of SFN against LNCaP cells, morphological changes were observed under an inverted microscope. The dead cell morphology with swelling and apoptotic shrinkage were observed in SFN-treated LNCaP cells as compared with untreated control (Figure 1B).

**Figure 2:**
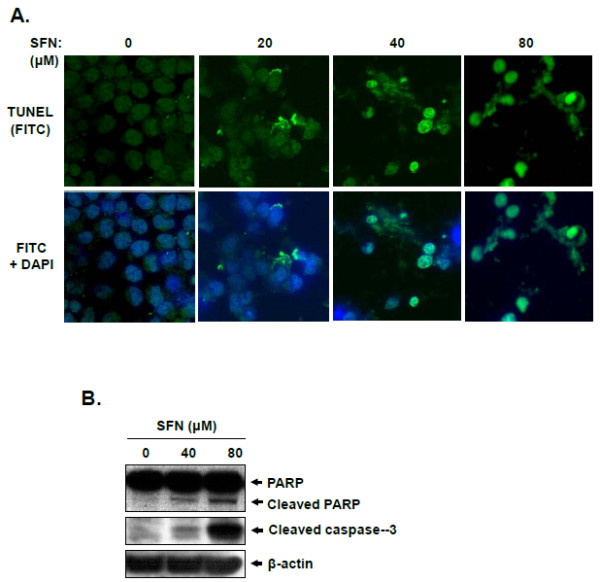
**Effect of sulforaphane on apoptosis induction in LNCaP cells**. (A) TUNEL assay was carried out in LNCaP cells treated with or without SFN (80 μM) for 24 h and fluorescence images were visualized under an Axio vision 4.0 fluorescence microscope (×630). (B) Cells were treated with SFN (0, 40 or 80 μM) for 24 h. Cell lysates were prepared and subjected to Western blotting for PARP and cleaved caspase-3.

To assess whether the cytotoxic property of SFN against LNCaP cells is linked to apoptosis, TUNEL assay was carried out. As shown in Figure 2A, SFN increased TUNEL positive cells in a concentration-dependent manner compared with untreated control. Further, SFN clearly induced the cleavages of PARP as well as caspase-3 (Figure 2B). These results indicate that SFN induces apoptotic cell death in LNCaP cells.

### Differential protein profiles of SFN-treated and -untreated LNCaP cells

**Figure 3:**
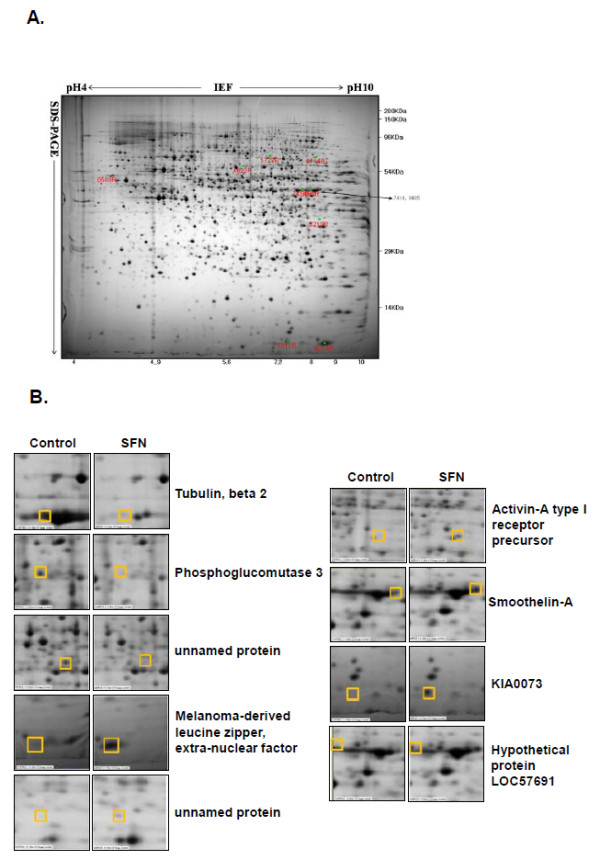
**Effect of sulforaphane on protein expression by proteomics analysis**. (A) Representative silver nitrate staining pattern in SFN (80 μM) treated LNCaP cells. Proteins (200 μg) were separated on pH 4-10 nonlinear IPG strip in the first dimension and 12% homogenous SDS-PAGE in the second dimension. Staining was performed with alkaline silver. (B) Gel image regions of SFN-treated group and untreated control with differentially expressed protein spots identified by Ettan MALDI-TOF analysis.

To understand the molecular mechanisms underlying SFN-induced apoptosis in LNCaP cells, 2-DE and gel sliver stain were conducted to analyze the changes in protein expressions. Representative 2-DE gel images for SFN-treated and control cells are shown in Figure 3A. Gel images were analyzed via PD-Quest software and detected about 1800 protein spots with pI between 3 and 10, and 300 protein spots were found to show the significantly different expressions between untreated control and SFN-treated cells (p < 0.05). These spots were cut from the gels and further identified by Ettan MALDI-TOF MS/MS analysis. Finally, we identified 9 protein spots exhibiting over 8-fold increase or decrease in abundance in replicate gels: tubulin beta-2 (spot 509), phosphoglucomutase 3 (PGM3) (spot 4620), unnamed protein product (spot 5728), smoothelin-A (spot 6011), KIAA0073 (spot 7414), Melanoma-derived leucine zipper, extra-nuclear factor (spot 8010), unnamed protein product (spot 8210), hypothetical protein LOC57691 (spot 8405), activin A type I receptor precursor (spot 8714) (Figure 3B and Table 1).

**Table 1:** Effect of SFN on the significantly changed protein expressions

**Spot No**.	Protein name	**NCBI accession No**.	Mr	pI	SC (%)	Expression of SFN-treated LNCaP cells
509	Tubulin, beta 2	NP_006079	52.68	4.37	35	decrease

4620	Phosphoglucomutase 3	CAI22635	55.93	5.8	22	decrease

5728	unnamed protein product	BAB85079	59.59	6.3	26	decrease

8010	Melanoma-derived leucine zipper, extra-nuclear factor	AAH63595	57.93	5.5	9	decrease

8210	unnamed protein product	BAC03859	39.90	4.6	20	increase

8714	Activin-A type I receptor precursor	NP_001096	58.33	7.2	11	increase

6011	Smoothelin-A	AAF03563	50.84	9.9	17	induce

7414	KIA0073	BAA07555	74.00	6.7	10	induce

8405	hypothetical protein LOC57691	NP_065982	90.63	8.1	10	induce

SC (%): Sequence coverage (%)

### Phosphoglucomutase 3 is involved in SFN-induced apoptosis in LNCaP cells

**Figure 4:**
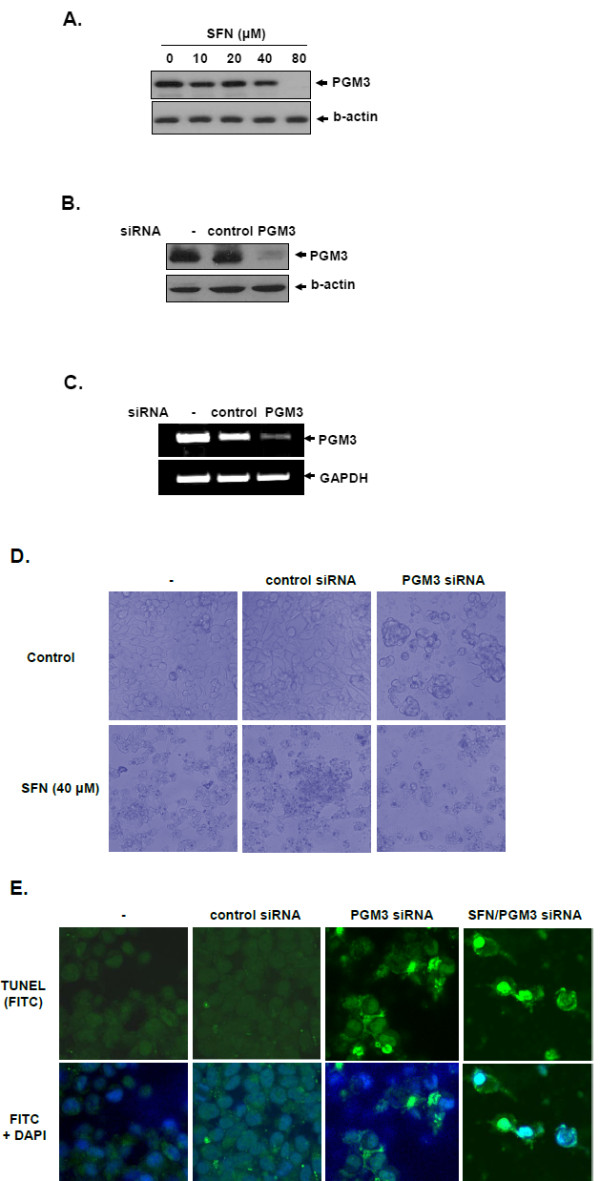
**Role of PGM3 in sulforphane-induced apoptosis in LNCaP cells**. (A) Effect of SFN on the expression of PGM3 protein in LNCaP cells treated with SFN (0, 20, 40 or 80 μM), examined by Western blotting. (B and C) Cells were transiently transfected with control or PGM3 siRNA. PGM3 expression was analyzed at protein and mRNA levels by Western blotting (B) and RT-PCR (C), respectively. (D) Effect of PGM3 siRNA on cytotoxic morphological changes in LNCaP cells treated with or without SFN. (E) TUNEL assay in PGM3 transfected LNCaP cells with or without SFN (40 μM) under an Axio vision 4.0 fluorescence microscope (×630).

Among nine identified proteins, PGM3 was chosen to further investigate its role in SFN-induced apoptosis in LNCaP cells. Cells were treated with various concentrations of SFN (0, 20, 40 or 80 μM) for 24 h and subjected to Western blot analysis with anti-PGM3 antibody. SFN modestly reduced the level of PGM3 between 10 μM and 40 μM and completely suppressed PGM3 at 80 μM. (Figure 4A).

To examine whether PGM3 is related to SFN-induced apoptosis in LNCaP cells, cell death was analyzed in LNCaP cells transfected with PGM3 siRNA in the absence or presence of SFN. Western blotting shown in Figure 4B revealed that PGM3 siRNA clearly suppressed PGM3 protein expression, while control siRNA did not affect PGM3 expression. Also, PGM3 siRNA transfection effectively inhibited the growth of LNCaP cells compared with siRNA control (Figure 4C). As shown in Figure 4D, morphological changes and growth inhibition were observed in LNCaP cells transfected by PGM3 siRNA under inverted microscopy. Furthermore, co-treatment with SFN and PGM3 siRNA synergistically increased the number of TUNEL-positive cells (Figure 4E), suggesting that PGM3 may be involved in mediating SFN-induced cell death in LNCaP cells.

## Discussion

Foods of cruciferous vegetables such as broccoli, cabbage, and kale are known for their anti-cancer activities against various types of cancers [13-17]. The anti-cancer effects of cruciferous vegetables were reported to be mainly due to the high levels of glucosinolates, which, following hydrolysis by the enzyme myrosinase, result in the formation of isothiocyanates [10,16]. Of the isothiocynates, SFN is an effective chemoprotective agent in carcinogen-induced animal models [18-20], and xenograft models of prostate cancer [21]. Recent works also showed the multifunctional action of SFN as Phase 2 enzyme inducer, cell cycle arrest and apoptosis [22-26].

Prostate cancer is the second leading cause of cancer-related death in men. Prostate adenocarcinoma is considered a disease of older men. However, almost one third of these men harbor evidence of the disease (high-grade prostatic intraepithelial neoplasia) between the age of 30 and 40 years. Epidemiological studies suggest that cruciferous vegetable intake may lower overall risk of prostate cancer, particularly during the early stages [27-30]; hence there are growing interests in identifying the specific chemopreventive agents from medicinal plants and in elucidating their mechanisms.

There are evidences that SFN can be a cancer preventive candidate for prostate cancer treatment. Herman-Antosiewicz et al. demonstrated that SFN-induced p21 protein protects against SFN-induced mitotic arrest in LNCaP prostate cancer cells [31]. Cho et al. demonstrated that SFN mediated the activation of c-Jun N-terminal kinase and caspase-mediated apoptosis in DU145 prostate cancer cells [11]. Nonetheless, the mechanisms of SFN in LNCaP prostate cancer cells still remain unveiled. Thus, in the present study, we investigated anti-cancer mechanisms of SFN in LNCaP cells by protemic analysis. SFN exerted cytotoxicity against LNCaP cells in a concentration-dependent manner with IC_50 _of ~62.5 μM (Figure 2A). Consistent with the results of previous reports [12,32], SFN increased DNA fragmentation, a hallmark of apoptosis, in TUNEL assay (Figure 2C) and cleaved PARP and activated caspase-3 (data not shown), suggesting the apoptotic effects of SFN in LNCaP cells.

Proteomics is the high-throughtput, large-scale, mainly automated analysis of protein mixtures to determine the expression level and post-translational modification of the proteins [33]. Proteomic analysis was performed to evaluate the mechanism of SFN-induced cell death in LNCaP cells. Nine protein spots from a total of ~1800 protein spots were selected for further study due to their most significant changes between SFN-treated group and untreated control in LNCaP cells. Three proteins were significantly down-regulated, including tubulin β-2 (NP_006079), phosphoglucomutase 3 (CAI22635) and unnamed protein product (BAB85079). In contrast, six proteins were significantly up-regulated, including melanoma-derived leucine zipper, extra-nuclear factor (AAH63595), activin A type I receptor precursor (NP_001096), smoothelin-A (AAF03563), KIAA0073 (BAA07555), hypothetical protein LOC57691 (NP_065982) and unnamed protein product (BAC03859) (Figure 3B and Table 1).

Of these proteins, tubulin β-2, a member of tubulin protein which is a major component of the cytoskeleton, was known to be targeted in chemotherapeutic agents such as taxane and Vinca alkaloid-induced apoptosis in cancer cells [34,35]. Smoothelin is a cytoskeletal protein which is generally found in smooth muscle cells [36] and consists of isoforms smoothelin-A and -B. Smoothelin-A was reported to be increased in expression in smooth muscle cells in apoptosis induced by pre-B-cell colony- enhancing factor (PBEF) [37]. These reports are consistent with our proteomics observations on these two proteins, and also support our proteomics identification of PGM3 as a mediator of apoptosis.

PGM3 is a member of the hexose-phosphate mutase family and essentially functions in glycogenolysis and glycogenesis [38]. In normal prostate cells, androgen withdrawal induces apoptosis and develops to prostate cancer [39]. However, considering that androgen metabolism is associated with the regulation of glycolytic enzymes and pentose-phosphate pathway [40], Pang's report that PGM3 converts glucose-1-phosphate to glucose-6-phosphate and mediates glycolysis as well as pentose phosphate shunt [38] suggests the possible involvement of PGM3 in prostate cancer metabolism. In light of these events, we considered that PGM3 may play a role in the regulation of prostate cancer cell survival. Our Western blotting results demonstrated that SFN significantly decreased the expression of PGM3 in a concentration-dependent manner in LNCaP cells and clearly suppressed at 80 μM of concentration (Figure 4A). According to Gasper and Pledgie-Tracy's studies, SFN can reach at high concentration to human prostate tissues physiologically and be restrained in various tissues including prostate [41,42]. Consistent with the results of Western blotting, TUNEL assay revealed that PGM3 siRNA increased green fluorescence TUNEL positive cells and also enhanced SFN induced apoptosis compared with control siRNA (Figure 4D). These data and previous reports suggest that PGM3 mediates SFN induced cell death in LNCaP cells, possibly via cancer metabolism.

## Conclusions

In summary, SFN effectively activated caspase-3 and increased TUNEL positive cells in LNCaP cells. Using proteomics analysis, we have identified nine proteins that might be involved in SFN induced apoptosis in LNCaP cells. Among them, we have verified SFN-reduced PGM3 expression, and demonstrated that PGM3 siRNA enhanced cytotoxicity and increased TUNEL positive cells in LNCaP cells compared to control. Our findings suggest that PGM3 is involved in mediating SFN-induced cell death in LNCaP cells and may be a potential molecular target for prostate cancer therapeutics.

## Methods

### Materials

_L_-Sulforaphane (purity ≥ 98%) (Figure 1), urea, thiourea, 3-[(3-cholamidopropy) dimethyammonio]-1-propanesulfonate (CHAPS), dithiothreitol (DTT), benzamidine, Bradford solution, acrylamide, iodoacetamide, bis-acrylamide, sodium dodecyl sulphate (SDS), acetonitrile, trifluoroacetic acid and α-cyano-4-hydroxycinnamic acid were purchased from Sigma-Aldrich Co. (St. Louis, MO). Pharmalyte (pH 3.5-10), IPG DryStrips (pH 4-10 NL, 24 cm) and Modified porcine trypsin (sequencing grade) were from Amersham Biosciences, Genomine Inc. and Promega (Maison, WI), respectively.

### Cell culture

LNCaP cells were purchased from the American Type Culture Collection (ATCC) (Manassas, VA) and maintained in RPMI 1640 supplemented with 10% fetal bovine serum (Welgene, Korea), 2 mmol/L L-glutamine, 10 mmol/L HEPES, 1 mmol/L sodium pyruvate and 45 g/L glucose with antibiotics at 37°C in a humidified atmosphere containing 5% CO_2_.

### Cytotoxicity assay

The cytotoxicity of SFN was assessed by XTT assay as described previously [43]. Cells were seeded onto 96-well microplates at a density of 1 × 10^4 ^cells per well in 100 μl of RPMI 1640 medium and grown for 24 h. Then the cells were exposed to various concentrations (0, 25, 50 or 100 μM) of SFN in serum-free medium for 24 h and 50 μl of XTT (1 mg/ml in phosphate buffered saline (PBS)) mixture containing phenazine methosulfate (PMS) (1.53 mg/ml in PBS) at the ratio of 100:1 was added to the cells. Cells were incubated at 37°C for 2 h and the optical density was measured using microplate reader (Molecular Devices Co.) at 450 nm. Cell viability was calculated as a percentage of viable cells in SFN-treated group versus untreated control using the following equation:

Cell viability (%)=[OD (SFN)−OD (blank)/OD (control)−OD (blank)]×100.

### Two-dimensional polyacrylamide gel electrophoresis (2DE-PAGE)

Cells were treated with or without SFN (80 μM) for 24 h, homogenized using mortor-driven homogenizer (PowerGen125, Fisher Scientific) in lysis buffer (7 M urea, 2 M thiourea containing 4% CHAPS, 1% DTT and 2% pharmalyte, and 1 mM benzamidine) and incubated for 1 h at room temperature. After centrifugation at 15,000× g for 1 h at 15°C, soluble fraction was collected and used for two-dimensional gel electrophoresis. Protein concentration was measured by the Bradford assay.

The first-dimensional isoelectric focusing (IEF) was carried out on Pharmacia Immobiline IPG DryStrip system (Uppsala, Sweden). For the first dimension of electrophoresis, the samples containing 100 μg protein for analysis gels were diluted to 350 μL with a rehydration solution (7 M urea, 2% w/v CHAPS, 50 mM DTT, 0.5% v/v IPG buffer (pH 3-10 nonlinear and pH 4-7 linear), and trace bromophenol blue) before loading onto 17 cm IPG strips (pH 3-10 nonlinear and pH 4-7 linear). IEF was then performed using IPG electrophoresis unit according to the manufacturer's instructions. Thereafter, the strips were equilibrated with a solution (6 M urea, 30% v/v glycerol, 2% w/v SDS, and 50 mM Tris-HCl, pH 8.8), reduced with 1% w/v DTT for 15 min, and alkylated with 2.5% w/v iodoacetamide for 15 min. Strips were then rinsed in electrophoresis buffer (25 mM Tris base, 192 mM glycine, and 0.1% w/v SDS), applied to 11% acrylamide gels, and sealed with melted agarose (0.5% w/v agarose in electrophoresis buffer containing a trace of bromophenol blue). SDS-PAGE was carried out using Hoefer SE 600 vertical chambers and a Tris-glycine buffer (25 mM Tris and 192 mM glycine) containing 0.1% w/v SDS, with initial separation at a constant 10 mA/gel for 30 min followed by 20 mA/gel. The second-dimensional SDS-PAGE was developed until the bromophenol blue dye marker had reached the bottom of the gel. The total run time was typically 4 to 4.5 hours. Gels were fixed in 10% v/v acetic acid, 40% v/v ethanol before sensitization for 30 min in a buffer containing 30% v/v ethanol, 0.2% w/v sodium thiosulphate, and 0.83 M sodium acetate. This was followed by three 15 min washes in deionised water. The proteins were then stained with 0.1% w/v silver nitrate for 20 min, washed twice in deionised water for 1 min, and developed in 2.5% w/v sodium carbonate containing 0.04% v/v formaldehyde (37% solution). The development was stopped with 1% v/v acetic acid, and the gels were washed three times in water.

### Image analysis

Quantitative analysis of digitized images was carried out using the PDQuest (version 7.0, BioRad) software according to the protocols provided by the manufacturer. Quantity of each spot was normalized by total valid spot intensity. Protein spots with intensity changed over two folds compared with control samples were selected for further studies.

### Enzymatic digestion of protein in-gel

Protein spots were enzymatically digested in-gel similar to that described by Shevchenko et al [44] but modified using porcine trypsin. Gel pieces were washed with 50% acetonitrile to remove SDS, salt and stain, dried to remove solvent and then rehydrated in Trypsin (8-10 ng/μl) Digest Solution and incubated 8-10 h at 37°C. The proteolytic reaction was terminated by addition of 5 μl 0.5% trifluoroacetic acid. Tryptic peptides were recovered by combining the aqueous phase from several extractions of gel pieces with 50% aqueous acetonitrile. After concentration the peptide mixture was desalted using C_18_ZipTips (Millipore), and peptides eluted in 1 to 5 μl of acetonitrile. An aliquot of this solution was mixed with an equal volume of a saturated solution of a-cyano-4-hydroxycinnamic acid in 50% aqueous acetonitrile, and 1 μl of mixture spotted onto a target plate.

### MALDI-TOF analysis and database search

Protein analysis was performed using an Ettan MALDI-TOF (Amersham Biosciences). Peptides were evaporated with a N2 laser at 337 nm, and using a delayed extraction approach. They were accelerated with 20 Kv injection pulse for time of flight analysis. Each spectrum was the cumulative average of 300 laser shots. The search program ProFound, developed by The Rockefeller University http://129.85.19.192/profound_bin/WebProFound.exe was used for protein identification by peptide mass fingerprinting. Spectra were calibrated with trypsin auto-digestion ion peak m/z (842.510, 2211.1046) as internal standards.

### Western blotting

Cell lysates were prepared by using lysis buffer (50 mM Tris-HCl, pH 7.4, 150 mM NaCl, 1% Triton X-100, 0.1% SDS, 1 mM EDTA, 1 mM Na_3_VO_4_, 1 mM NaF, 1× protease inhibitor cocktail). The extracts were incubated on ice, spun at 14,000× g for 20 min at 4°C and the supernatants were collected. Protein concentrations were determined by Bradford assay (Bio-Rad), and the protein (50-100 μg) was separated by electrophoresis on 4-12% NuPAGE Bis-Tris gels (Novex). Proteins were then transferred to Hybond ECL transfer membranes to evaluate their expression using PGM3 (Santa Cruz Biotechnologies) and β-actin (Sigma) antibodies.

### TUNEL assay

Apoptotic cell death was examined by using DeadEnd™fluorometric TUNEL assay kit as described in the manufacturer's instructions. Briefly, cells were plated onto the poly-_L_-lysine-coated slides, fixed with 4% paraformaldehyde for 15 min and incubated in TdT enzyme buffer containing fluorescein-12-dUTP for 1 h at 37°C. After mounting in medium containing DAPI (Vectashield, Vector Labs), cells were visualized under a Carl Zeiss LSM5 confocal microscope.

### siRNA transfection

Cells were transiently transfected with PGM3 or control siRNA (Santa Cruz Biotechnology, Santa Cruz, CA) at 50 nM of final concentration by using INTERFERin siRNA transfection reagent (Polyplus transfection) for 72 h.

### RT-PCR

Total RNA was prepared using the Trizol reagent (Invitorgen) following the manufacturer's instructions and reverse transcribed to cDNA using oligo-dT and random primers. The cDNA was amplified by PCR using the following specific primers:

PGM3: forward 5'-ACACGCCAAGCCCAATGGACT- 3',

reverse 5'-TTCTCACTGCTGGGCCTGGT-3';

GAPDH: forward 5'-TCACCATCTTCCAGGAGCGA-3'

reverse 5'-CACAATGCCGAAGTGGTCGT-3'

PCR conditions were as follows: 92°C for 2 min; 94°C for 30 sec, 59°C for 30 sec, 72°C for 30 sec (30 cycles); and 72°C for 5 min. The amplified products were separated on 2% agarose gels.

### Statistical Analysis

All data were expressed as means ± SD. The statistically significant differences between control and SFN treated group were calculated by the Student's t- test.

## Competing interests

The authors declare that they have no competing interests.

## Authors' contributions

Conceived and designed the experiments: CL, SJ, KA, HL, KA, SK. Performed the experiments: CL, SJ, SM, JK, HL, HK. Analyzed the data: CL, SJ, HL, KA, SK. Wrote the paper: SJ, KA, SZ, CC, SK. All authors read and approved the final manuscript.

## Supplementary Material

Additional file 1 **Figure S1. Chemical structure of sulforaphane (SFN)**. Molecular weight = 177. Click here for file
