# Supplementary figures and images for: Down-regulation of phosphoglucomutase 3 mediates sulforaphane-induced cell death in LNCaP prostate cancer cells
Source: Proteome Sci. 2010 Dec 16;8:67. doi: 10.1186/1477-5956-8-67 (PMC3024296; doi:10.1186/1477-5956-8-67)

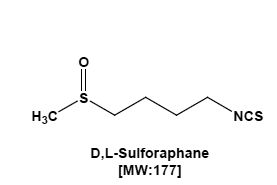

Supplement: Additional file 1 — Figure S1. Chemical structure of sulforaphane (SFN). Molecular weight = 177. [file 1477-5956-8-67-S1.TIFF]
